# Size Dependent Male Reproductive Tactic in the Two-Spotted Goby (*Gobiusculus flavescens*)

**DOI:** 10.1371/journal.pone.0143487

**Published:** 2015-12-07

**Authors:** A. C. Utne-Palm, K. Eduard, K. H. Jensen, I. Mayer, P. J. Jakobsen

**Affiliations:** 1 Institute of Marine Research, Bergen, Norway; 2 Department of Biology, University of Bergen, Bergen, Norway; 3 Norwegian University of Life Sciences, Oslo, Norway; 4 Oslo University Hospital, Oslo, Norway; University of Padova, ITALY

## Abstract

Male investment in testes and sperm duct gland in the polygamous nest breeding two-spotted goby *Gobiusculus flavescens* (Fabricius) was investigated in relation to time in reproductive season and individual physical parameters. This small teleost fish is most likely the most abundant species found along the rocky shores of the North East Atlantic. The two-spotted goby has a single reproductive season, during which nest-caring males can raise several clutches of offspring. According to the literature the males are on average larger than the females. Here we report for the first time a population showing a reversal of this trend, with males on average being smaller than females, a difference likely caused by a large proportion of small males. Early in the breeding season these small males have typical sneaker characters, with relatively large testes and small seminal duct glands compared to the larger dominant territorial males. The presence of these two alternative male reproductive tactics is confirmed by histological studies, which shows the presence of sperm in the sperm duct glands (SDG) of smaller males, but not in the SDG of intermediate and larger males. To our knowledge, males with typical sneaker characters have not been reported in earlier studied populations of two-spotted goby. Interestingly we found that testes investment declined significantly over the course of the breeding season, and that this reduction was significantly more pronounced in small compared to the large males. Further, a significant increase in seminal duct gland (SDG) mass was observed for the smaller males over the breeding season. We propose that this indicates a possible shift in mating tactic by smaller males from a parasitic to a nest-holding tactic over the course of the breeding season. Thus, the observed size dependent plasticity in investment in SDG over time suggests that the reproductive tactic of *G*. *flavescens* is conditional, and possibly influenced by mate availability and male—male competition.

## Introduction

Competition for mating partners may lead to alternative mating strategies and tactics [1]. In teleost fishes in particular, this is often manifested in the occurrence of two viable alternative male reproductive strategies. The larger dominant males strive to monopolise mates by a combination of elevated aggressive behaviour and an increased investment in secondary sexual traits (nuptial colouration, ornaments and chemical signals), and/or by monopolising resources important to the female. Alternatively, the generally smaller males compete with the dominant males by adopting a parasitic tactic, either by sneaking behaviour or female mimicry, where individuals avoid making such investments and instead, exploit the reproductive investment of others. By saving costs related to the development of secondary sexual traits, mate attraction and intra-sexual contests, parasitic spawners (sneakers) can alternatively invest more in sperm production [2– 4]. However, only in a few cases is the choice of male reproductive tactic fixed for life, and in most cases it is plastic, being conditional on the prevailing conditions which will determine which of the alternative male reproductive strategies will increase fitness most [5]. The decision on which mating tactic to adopt is usually correlated to body size [5], and commonly an ontogenetic transition from a parasitic to a dominant tactic occurs through life as the male grows [4, 6]. The tactic switch point should be sensitive to both ecological and demographic events—as they both can affect the fitness of a given tactic [5]. In species where males are using conditional reproductive strategies such as physical condition, status (relative size), population density [7] or sex ratio [6, 8, 9, 10, 11], all these factors have been found to influence the male’s choice of tactic.

In common with most teleost fishes displaying alternative mating tactics, a conditional tactic seems to be most common in species of the family Gobidae. In gobies, larger males usually adopt the dominant mating tactic (territorial nest holders), while the smaller males adopt the parasitic tactic (sneakers) [12–14]. Even more drastically, some gobies change their sex and hence their reproductive tactic completely [15].

**Fig 1:**
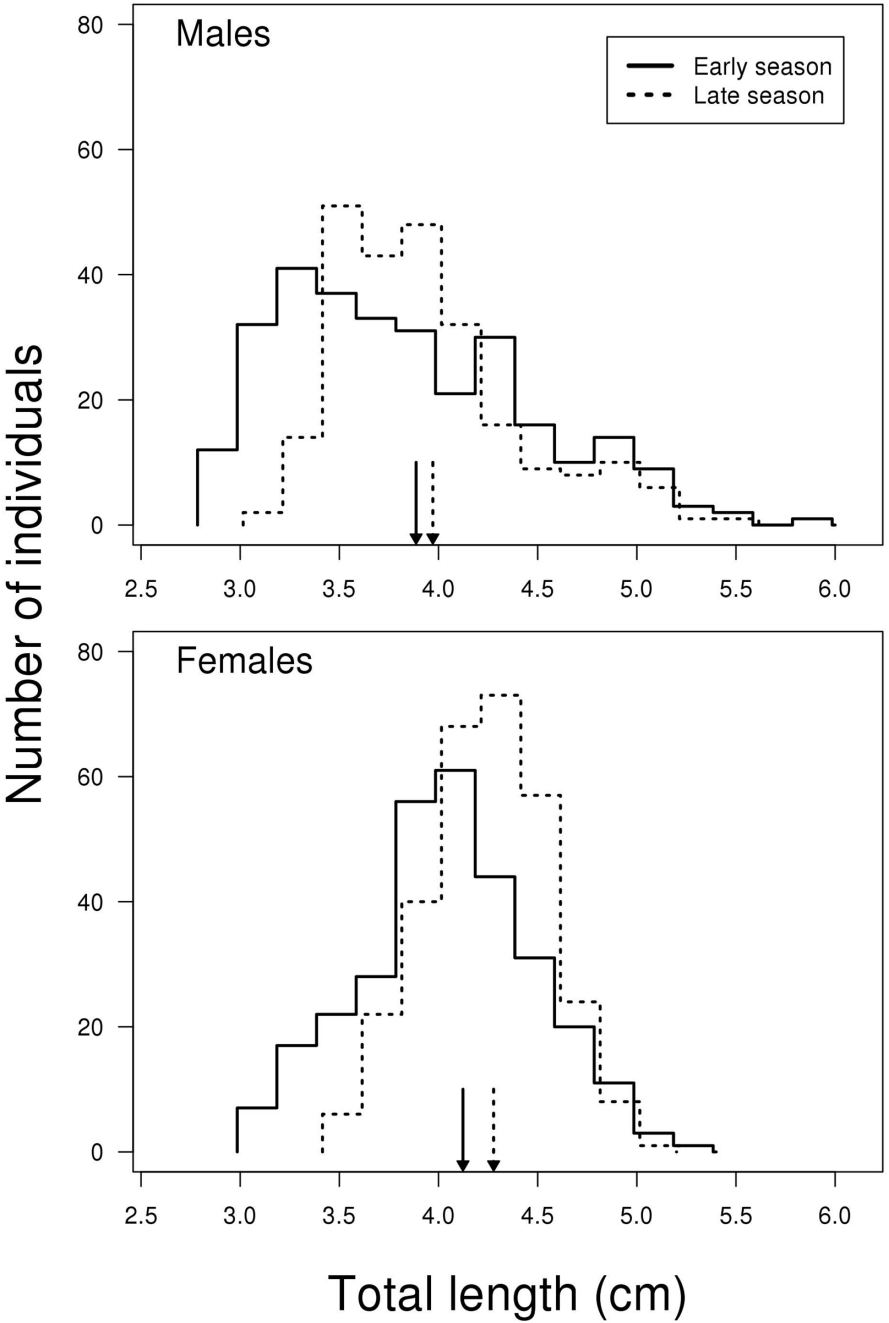
Size distribution of male and female two-spotted goby in early (May) and late (July) reproductive season. To make it easier to distinguish between the lines in cases where they overlap, the lines for early and late season are placed 0.015 units to the left and right on the x-axis, respectively. Average lengths related to season are marked with arrows. Sample size females N = 600, males N = 533.

In the Bergen area (south-west Norway) the two-spotted goby *Gobiusculus flavescens* (Fabricius) are born in May to August, they spawn the following season and die during fall after their one and only spawning season (become ca 1.5 years old) [16]. However, during this single reproductive season males can mate—and care for several clutches of offspring [17]. The males are known to adopt a nest in indentations on rocky surfaces, in empty mussel shells or in the hold fast of kelp, from where they attract females. According to earlier studies in Ireland [18], Scotland [17],and Sweden [19] the two-spotted goby exhibits sexual size dimorphism, where males are on average larger than females. However, in the presented studied population on the west Norwegian coast (Bergen) this size dimorphism is reversed as average female size is slightly larger than average male size, caused by a large proportion of very small males (Fig 1). Our prediction was that these small males might act as parasitic spawners (sneakers).

A study from the west coast of Sweden, [19] (where we find the traditionally reported size dimorphism ♂ > ♀) previously showed that over the short breeding season of the two-spotted goby there was a transition from a strong male–male competition with intensive courting males in the beginning of the season to a strong female–female competition with actively courting females towards the end of the season. This observation has recently been supported by further studies in the same location (Gullmarsfjorden, west coast of Sweden), reporting that i) the size of nest holding males decreased over the season—indicating a decrease in male—male competition [20], and ii) courtship is typically initiated by males and terminated by females early in the season, while the opposite pattern is found late in the season [21].

In the two-spotted goby, nest defence, brood care and courtship are energetically demanding [22], which probably causes increasing mortality rates of nest holding males over the season. Thus one should expect to find smaller males using a sneaking strategy, especially early in the breeding season when male—male competition is at its highest.

Nest holding male gobies are known to lay mucous ejaculates, defined as sperm trails, which slowly dilute into the water and release active sperm for several hours, reaching eggs via the surrounding water [23, 24]. The mucins of the ejaculate are produced by a pair of accessory sperm duct glands (SDGs), whose secretory activity have been found to vary in gobioid species where males perform alternative mating tactics [24, 25]. Large dominant males, which build nests and perform parental care, exhibit larger SDGs and more abundant secretions than males using a parasitic tactic [24, 25]. Further, it has been reported that parasitic spawners, and not dominant males, use their SDGs for sperm storage [25]. Thus, the presence and the relative development and use of SDGs represent an excellent proxy to evaluate the presence of alternative male mating tactics.

The present study was undertaken to determine firstly, whether the small males discovered in the Bergen population display a parasitic spawning tactic. Secondly, if so we wanted to determine whether the prevalence of this spawning tactic changes over the course of the breeding season.

Given the difficulty in finding nests in the field, and that small males are rarely observed (< 5% of all males observed when snorkelling were small males), our conclusions are based on physiological and histological findings from fish sampled by beach seining. Histological evaluation of SDG morphology should underpin the findings of a sneaking tactic among the smaller males, by revealing low mucin production and presence of sperm in the SDGs. As such, our conclusions will be made from SDG histology together with data on testes and SDG investment—across season and location. In earlier studies the flexibility in male tactics of a species has been demonstrated experimentally by manipulating the level of male-male competition or female availability i.e. [9, 12, 14, 26, 27]. Here we demonstrate seasonal plasticity in a natural un-manipulated population.

## Methods

### Studied species

The two-spotted goby is small ubiquitous marine fish (3–5 cm), which inhabits the sub-tidal area along rocky shores in the North East Atlantic (from Gibraltar to Northern Norway). The two-spotted goby is probably the most numerous littoral fish species found along rocky shores of North East Atlantic, where it reaches average densities of 70–165 individuals m^-3^ [16]. During the breeding season males occupy and defend nest sites, commonly located in the holdfast or blades of kelp (*Laminaria* spp.), indentations on rocky surfaces or empty mussel shells [28]. They have a single spawning season (in the present study area from May to August) during which both males and females spawn several times. Males of two-spotted gobies display pronounced parental care, with nest holding males guarding, fanning and cleaning the eggs until hatching [22]. A nest holding male continues to attract females throughout the season and can have eggs from several females at the time [17, 22, 29].

### Fish sampling and handling of samples

Fish samples were collected during the early (May) and late (July) breeding season in 2006 and 2008, using a beach seine (30 m long, 3 m high, mesh size 3–5 mm in the catch area). Some modifications were made to the seine to ensure that it followed the topography without lifting off the bottom by the macro algae; this included additional weights to the bottom line and floaters on the top line. To ensure that a true representative sample of nest holding males was collected (hiding in their nest on the bottom) one seine net was first set to effectively enclose our sampling area, after which the enclosed area was sampled twice with a second beach seine, before finally the outer seine was hauled in.

Three different sampling locations on the west coast of Norway were selected in order to obtain samples as representative as possible of the habitat of the two-spotted goby. These locations were the inner and outer Fanafjord and the Kvalen Raunefjord, all located north of Bergen, which differed in terms of exposure, temperature and salinity. Fish used in the histological studies where captured by beach seine in May and July 2008 at the Kvalen Raunefjord location.

To avoid stress and injury during capture the beach seine was pulled in slowly, and the fish were gently collected by dip net and placed into shaded buckets containing 20 litres of fresh seawater as well as some fresh kelp for shelter. From the seine samples, a random and representative subsample of 100 females and 100 males was taken (or less than 100 if less fish were caught in the beach seine), after which the remaining fish, as well as non-target species, were carefully returned to the sea. Females and males are easy to distinguish by eye due to distinct ornamentation during the reproductive season (males has iridescent blue spots and females has orange bellies) [19]. The small males also display these ornamental colours. Identification of sexes during the breeding season was determined by the marked differences in colour patterns and nuptial colouration between the sexes [28]. The sub-samples of gobies were quickly transported back to the aquaria facility at Espegrend field station, University of Bergen, where they were transferred to two 100 litre tanks in a temperature control room. The tanks were supplied with artificial kelp shelter and through-flowing aerated natural seawater kept under ambient photoperiod and the same temperature (13–18°C) as in the field. Later, on the same day of capture, all fish (one by one) were netted out with a dip net to be sacrificed by an overdose of MS222 (3-aminobenzoic acid ethyl ester). Each fish was individually weighed (± 0.1 mg) and measured (total length; ± 1.0 mm), after which the fish was dissected to confirm sex. For males, the paired testes and SDGs were carefully separated and weighed individually in order to calculate the gonadosomatic index (GSI = testes weight + SGD weight/body weight x 100) and seminal gland duct index (SGDI = SGD weight/body weight x 100). The GSI was also calculated for females. In addition, the soma body mass (body weight—gonad weight) and condition factor (CF = body weight (g) x 100/body length (cm)^3^ were calculated for both sexes. Only data of the male gonads are presented here.

### Histology

For gonad histology studies a size representative group of males (N = 18) were sampled from the May and the July seine catches. Fish were caught and handled as described above. Following dissection, testes and SDGs were excised, weighed separately (±0.1 mg), and fixed in Dietrich’s solution (900 ml distilled water, 450 ml 95% ethanol, 150 ml 40% formaldehyde, 30 ml acetic acid) for 4–5 months before being embedded in paraplast. Seminal vesicles and gonads were sectioned transversally (6 μm) and mounted on slides for histological examination. Sections were stained with Haematoxylin and eosin for general histology, and periodic acid-Schiff (PAS) a classical histochemical stain to identify polysaccharides [30]. Using the picture analysing program Image J we estimated the fractional area of lumina (indicating mucin production) and chamber wall for all SDGs, representing males of different size categories. To estimate the average real fraction we used the plug-in ObjectJ of ImageJ [31]. Here a fine resolution version of the standard Weibel multipurpose grid [32] was designed and used, consisting of 209 line fragments giving a total of up-to 418 hits per field. We picked three sections from different parts of the SDG to calculate the average fractional area of lumina and chamber wall for each fish. Further, the relative abundance of sperm in SDG was calculated by counting number of lumina within the three sections containing sperm divided by total number of lumina.

### Statistics

All statistics and plots were performed using R version 3.2.2 (R Development Core Team 2015, http://www.r-project.org). Fish size was compared between sex and season by using a linear mixed effects model (LME) with the predictor total length of the fish (cm) and the two categorical predictors season and sex. Station was set as a random effect factor. Sample sizes for males and females were 533 and 600, respectively. Demography was investigated by making simple frequency plots of fish lengths depending on sex and season. Additionally, for each sex we made size-frequency distributions over early and late season for the 25% smallest and largest fish, respectively. This allowed us to get a clearer picture of eventual demographic differences between males and females over the season.

For male fish, the investment in seminal duct gland (SDG) and testes were compared between early and late season, i.e, May vs July. We did this by using five different models to cover the most commonly used methods from earlier studies e.g. [24, 33].

*Model A*, *GSI*: The response variable was the gonadosomatic index (GSI) and the two predictors were total length and season.

*Model B*, *SDGI*: The response variable was the seminal duct gland index (SDGI) and the two predictors were total length and season.

*Model C*, *Testis mass*: The response variable was log (testis weight) and the predictors were log(soma mass) and season.

*Model D*, *SDG*: The response variable was log (seminal duct gland weight) and the two predictors were log (soma mass) and season.

*Model E*, *relative investment in SDG*: The response variable was log (seminal duct gland weight/testes weight) and the two predictors were log (soma mass) and season.

Since the five models above consist of a continuous response variable and two predictors where one is continuous and one is categorical, it is natural to think about an ANCOVA where the two regression lines for early and late season are compared. However, to account for an eventual curvature in the data we used polynomial modeling for the continuous predictor of each model. Log likelihood ratio tests (LRT) were used for determining the number of curvature parameters (polynomial order) for the continuous predictor. Since the data were collected over three different stations, we needed to account for the random effect of stations. We did this by using linear mixed effects models LME [34, 35]. Since LMEs with two predictors and a polynomial term gives a rather complex set of outputs, we only list the most important results for each of the five models and give the full list of output in the supporting information material (S1 File). In cases where the number of curvature parameters determined from the LRT gave polynomial models with an order higher than three, we replaced the model with a generalized additive model (GAM). We did this since the biological interpretation of parameter estimates becomes extremely difficult for such high order polynomial models.

In the models about SDG and testes investment the sample size was reduced from 533 to 506 as we were lacking SDG and testes measurements for 27 of the fish (due to problems separating the testes and SDG during dissection).

Condition factor was analysed for females and males in one model, allowing for a three way interaction between the predictors; total length of the fish, season and sex. We used an LME for this purpose and the random effect factor was the same as explained in the models above.

Based on histological findings on whether the SDGs contained sperm or not we divided the males into either “sneakers” (size range with sperm in SDG) and “territorial” males (size range without sperm in SDG) for early and late season. Finally, we performed a linear regression (log (Y) = log (a) + b*log (X), where Y refers to SDG and Testes weight and X to soma weight), to look at possible difference in “b” (slope) between groups (sneakers and territorial) and season [33, 36, 37].

### Ethics

All aspects of this study, including field sampling, fish transportation, and killing of fish (by overdose of MS222), were approved in advance by the Animal Care Committee at the University of Bergen. This committee closely follows the strict regulations of the European Commission directive for animal used for scientific purposes. Collection of fish along the Norwegian coast does not require permission. The field collections did not involve endangered or protected species.

## Results

### Demographic findings

The comparison of fish size depending on sex and season revealed no interaction between the two predictors, i.e. the change in size when going from early to late season is the same for the two sexes (LME; F_1, 1127_ = 1.385, P = 0.240). However, there was a significant effect of both sex and season, where the mean size of males is significantly smaller than females (LME; F_1, 1127_ = 98.185, P < 0.001, Fig 1) and mean size is significantly smaller in the early compared to the late breeding season (LME; F_1, 1127_ = 22.087, P < 0.001, Fig 1). Among all male fish sampled in this study, the ones belonging to the size range representing the 25% smallest males are more represented in the early compared to the late breeding season, with 100 and 33 small males sampled in the early and late breeding season, respectively. The same trend was also observed for the 25% largest males with 82 and 51 large males in the early and late breeding season, respectively. For females, the 25% smallest individuals were represented with 92 and 58 females sampled in the early and late breeding season, respectively, while the 25% largest females, were represented with 66 and 84 females sampled in the early and late breeding season, respectively. Thus, our results indicate a higher mortality or a different growth rate among larger males compared to females in the late breeding season.

### Gonadosomatic index (GSI)

**Fig 2:**
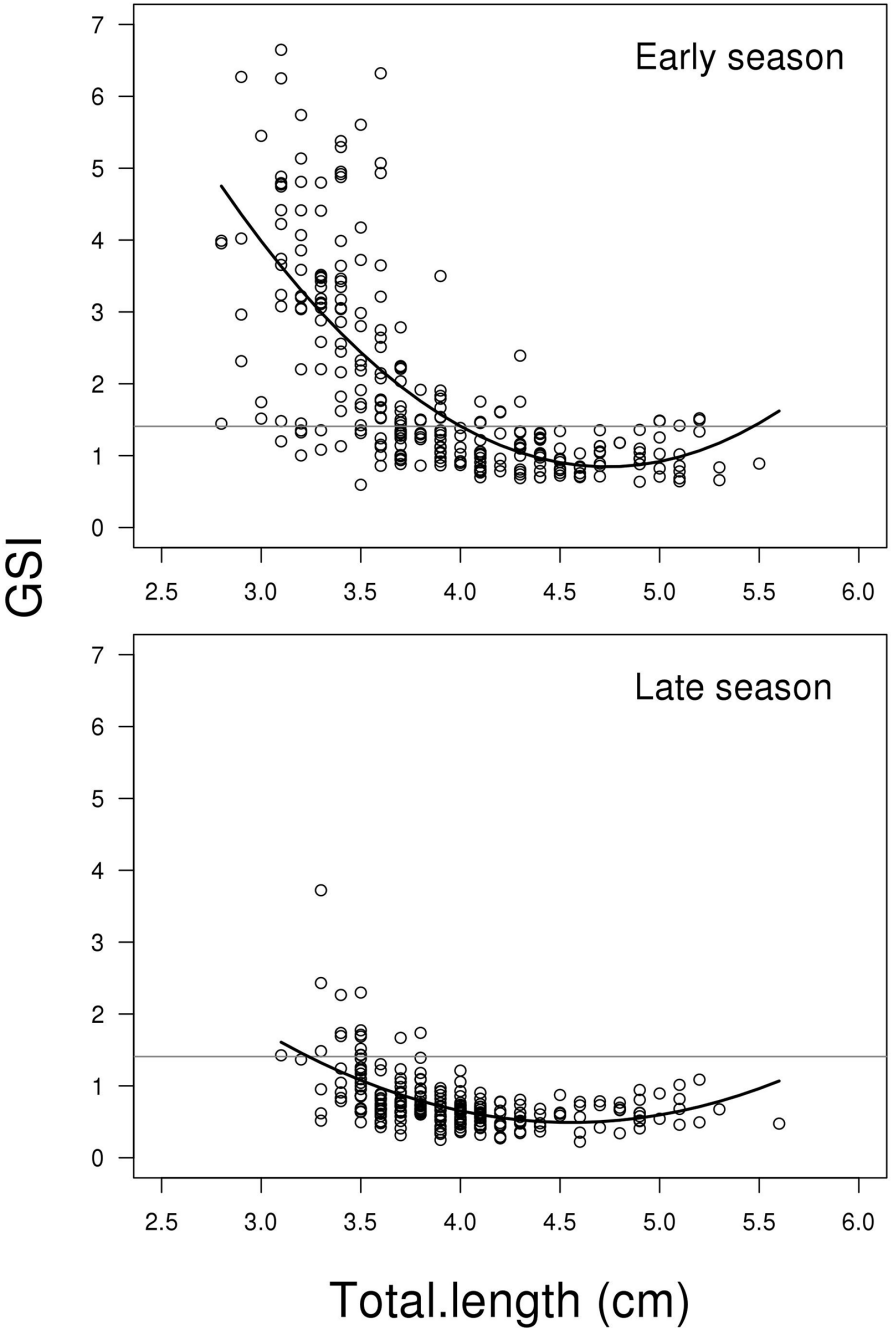
Scatterplots showing gonadosomatic index GSI (GSI = (testes weight + SDG weight) / body weight*100) related to total length for early and late reproductive season. The solid lines represent the mixed effect model. The grey horizontal lines represent the average of the full dataset—independent of season and soma mass. Early season has significant higher mean than late season and the relative decrease in GSI depending on total length is steeper in early compared to late season (for more details see results). N = 506.

The model for GSI showed a significant interaction between the two predictors Total length (including second order polynomial) and Season (LME; F_2,498_ = 21.763, P < 0.001, Fig 2). This indicates that the relationship between GSI and fish length changes from early to late in the breeding season. Fig 2 shows that this is mainly due to the high proportion of small fish having high GSI values in the early compared to the late breeding season. This is supported by the treatment contrasts from the model (the summary output of R): Early season has significantly higher mean than late season (contrast between mean of early and late season; t_498_ = 14.256, P < 0.001), and the relative decrease in GSI depending on total length is steeper in early compared to late season (contrasts between first order polynomials of early vs late season; t_498_ = 6.483, p < 0.001). The untransformed mean GSI in early and late season is 1.981 (SE = 0.084) and 0.778 (SE = 0.026), respectively. For a complete list of results from the model see Supporting information material (Model A in S1 File).

### Seminal duct gland index (SDGI)

**Fig 3:**
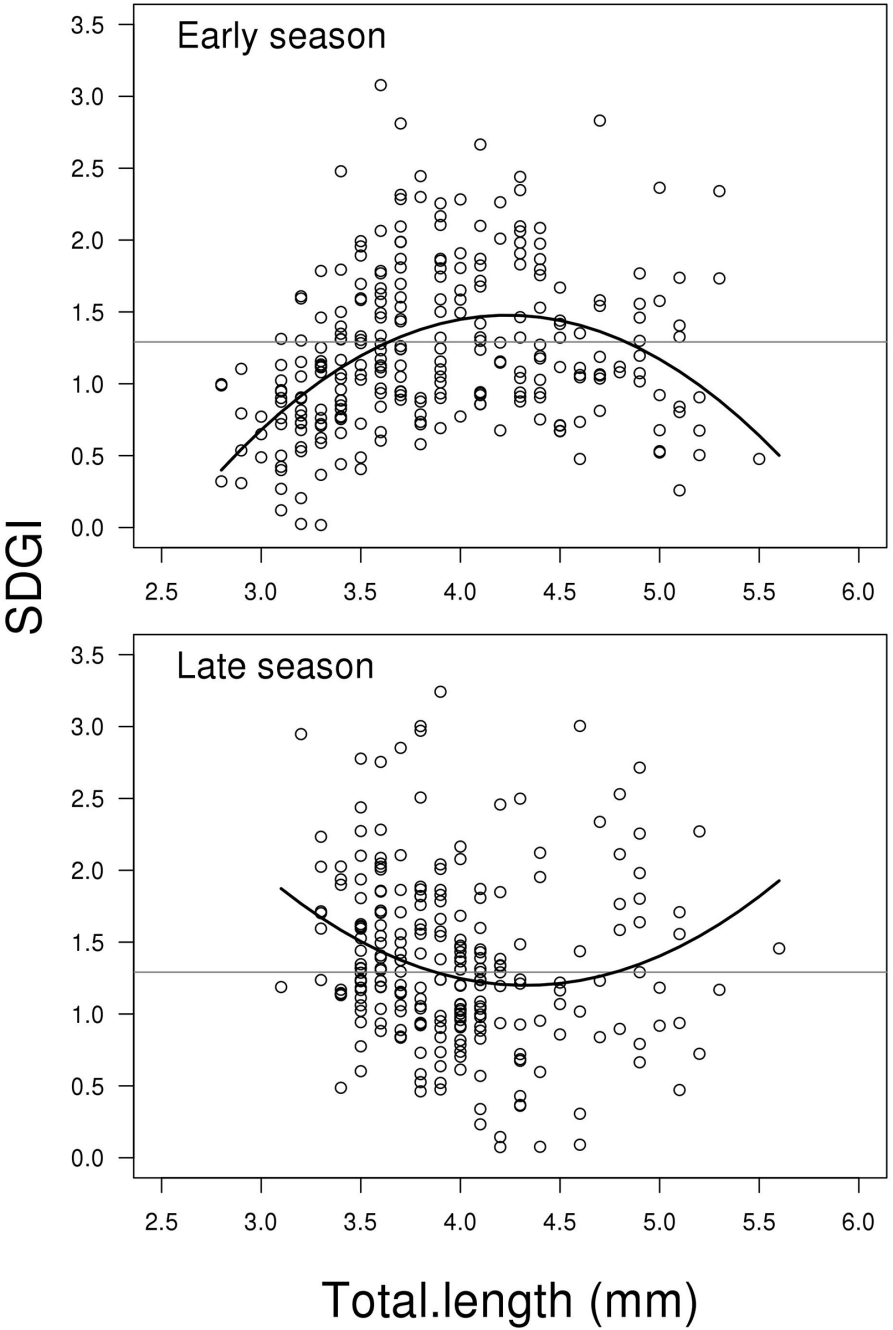
Scatterplots showing seminal duct gland index SDGI (SDGI = (SDG weight) / body weight*100) related to total length for early and late reproductive season. The solid lines represent the mixed effect model. The grey horizontal lines represent the average of the full dataset—independent of season and soma mass. Change in SDGI depending on fish length is different between early and late season. This is mainly due to opposite curvatures between early and late season data (for more details see results). N = 506.

The model for SDGI showed a significant interaction between the two predictors Total length (including second order polynomial) and Season (LME; F_2,498_ = 24.493, P < 0.001, Fig 3), which means that the change in SDGI depending on fish length is different between early and late season. Fig 3 shows that this is mainly due to opposite curvatures between early and late season data. Small males in the early season start out with a low SDGI that increases up to intermediate fish lengths and then declines again for the largest fish. In the late season, the curvature is the reverse. This difference in curvature is significant (contrast between second order polynomials of early vs late season; t_498_ = 6.434, P < 0.001, Fig 3). The mean SDGI-level in the early season is slightly lower than in the late season (t_498_ = 2.613, P = 0.009, Fig 3). For a complete list of results from the model see Supporting information material (Model B in S1 File).

### Testis weight

**Fig 4:**
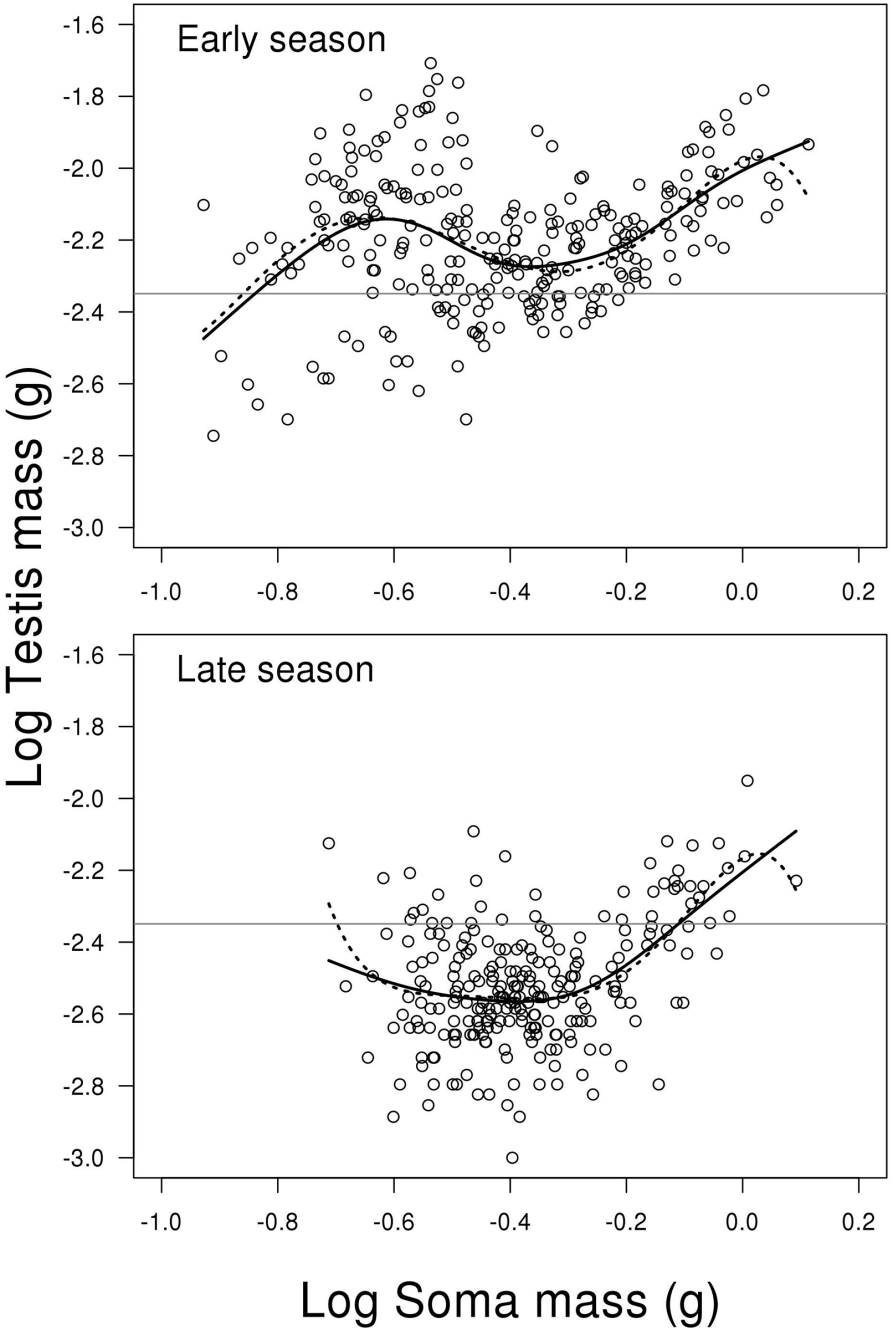
Scatterplots of log testes weight on log soma mass depending on reproductive season. The solid lines represent the generalized additive model, while the dotted lines represent the mixed effect model. The grey horizontal lines represent the average of the full dataset—independent of season and soma mass. The decline in testes weight for small fish when going from the early to late breeding season is more pronounced than for the large fish (see results for further details). N = 506.

The model for log testis weight showed a significant interaction between the two predictors log soma mass (including fifth order polynomial) and season (LME; F_2,492_ = 3.048, P < 0.010, Fig 4). This means that the change in testis weight depending on fish body mass is different between early and late season. Due to the high order polynomials, we replaced the model with a GAM, as shown in Supporting information material (Model C in S1 File). This model confirms that the effect of body mass depends on season, where there are more curvatures in early season (GAM; edf = 4.912, Ref. df. = 6.055, F = 9.741, P < 0.001, Fig 4), compared to late season (GAM; edf = 2.838, Ref. df. = 3.669, F = 9.433, P < 0.001, Fig 4). However, the most pronounced difference been early and late season is that the general level of investment is higher early compared to late in the season (GAM; contrast between mean of early and late season; t = 17.150, P < 0.001, Fig 4). The untransformed mean weight of testes in early and late season is 6.987 mg (SE = 0.195) and 3.302 mg (SE = 0.093), respectively. For a more complete list of results from the models see Supporting information material (Model C in S1 File).

### Seminal duct gland

**Fig 5:**
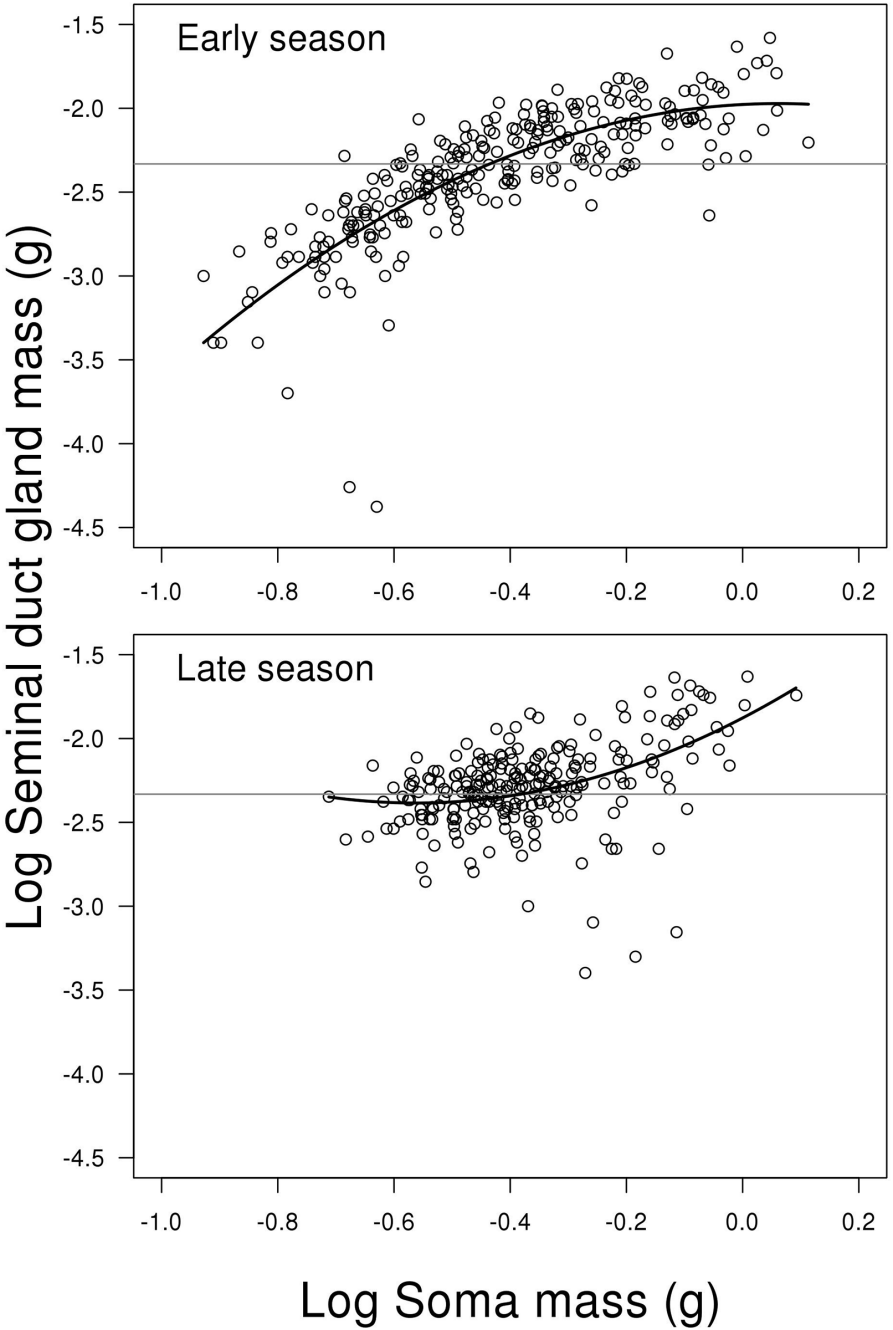
Scatterplots of log seminal duct gland mass on log soma mass depending on reproductive season. The solid lines represent the mixed effect model. The grey horizontal lines represent the average of the full dataset—independent of season and soma mass. Change in SDG investment depending on fish soma mass is different between early and late season. This is mainly due to a stronger positive relationship between relative investment in SDG and soma body mass in the early compared to late season (see results for further details). N = 506.

The model for SDG investment reveals a significant interaction between the two predictors log soma mass (including second order polynomial) and season (LME; F_2,498_ = 20.625, P < 0.001, Fig 5). This means that the change in SDG investment depending on fish soma mass is different between early and late season. Fig 5 and the summary output from R shows that this is mainly due to a stronger positive relationship between relative investment in SDG and soma body mass in the early compared to late season (contrast of first order polynomials between early and late season; t_498_ = 5.540, P < 0.001). The untransformed mean weight of seminal duct gland in early vs. late season is 5.711 mg (SE = 0.256) and 6.145 mg (SE = 0.250), respectively. For a complete list of results from the model see Supporting information material (Model D in S1 File).

### Relative investment in seminal duct gland

**Fig 6:**
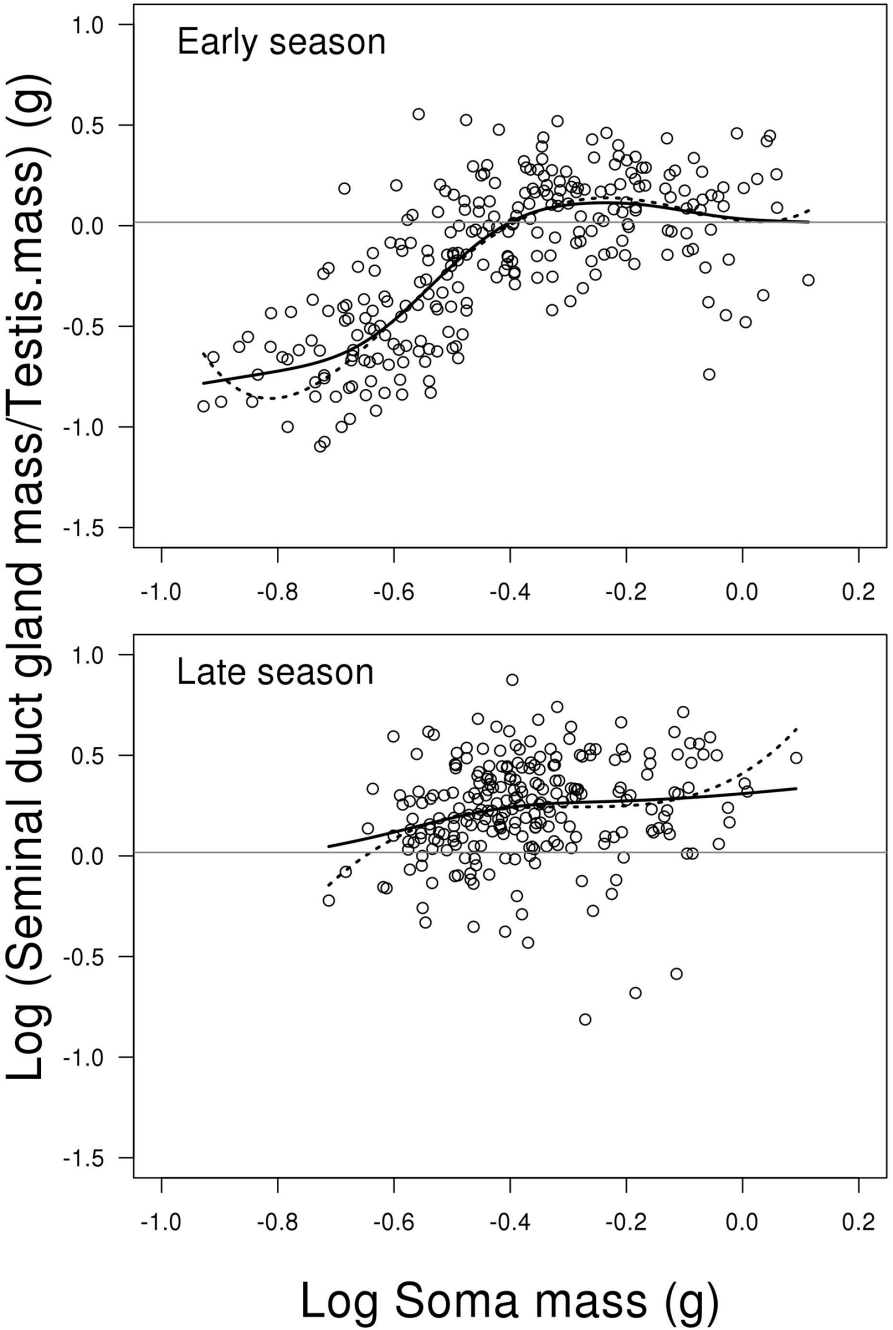
Scatterplots of log relative investment in seminal duct gland to testes weight on log soma mass depending on reproductive season. The solid lines represent the generalized additive model and the dotted lines represent the mixed effect model. The grey horizontal lines represent the average of the full dataset—independent of season and soma mass. Change in relative SDG investment depending on fish soma mass is different between early and late season. This is mainly due to a stronger positive relationship between relative investment in SDG and soma body mass in the early compared to late season. Further, relative SDG investment is lowest early season due to a low investment of fish with low soma mass (see results for further details). N = 506.

The model for relative investment in SDG, i.e. log (SDG weight/ testis weight), showed a significant interaction between the two predictors log soma mass (including fourth order polynomial) and season (LME; F_2,494_ = 9.989, P < 0.001, Fig 6). This means that the change in testis weight depending on fish body mass is different between early and late season. Due to the high order polynomials, we replaced the model with a GAM, as shown in Supporting information material (Model E in S1 File). This model confirms that the effect of body mass depends on season, where the curvature in early season is highly significant (GAM; edf = 3.999, Ref. df. = 5.008, F = 5.600, P = 0.001, Fig 6), while it is non-significant in the late season (GAM; edf = 0.667, Ref. df. = 0.667, F = 0.515, P = 0.558, Fig 6). This difference in curvature is seen from Fig 6, where the early season shows an increase in investment over increasing fish body mass that levels off for the biggest fish, while the increase over body mass is weaker in the late season (Fig 6). Further, the level of investment is lower early compared to late in the season (contrast between mean of early and late season; t = 12.200, P < 0.001, Fig 6). For a more complete list of results from the models, see Supporting information material (Model E in S1 File).

### Condition factor related to size, season and sex

**Fig 7:**
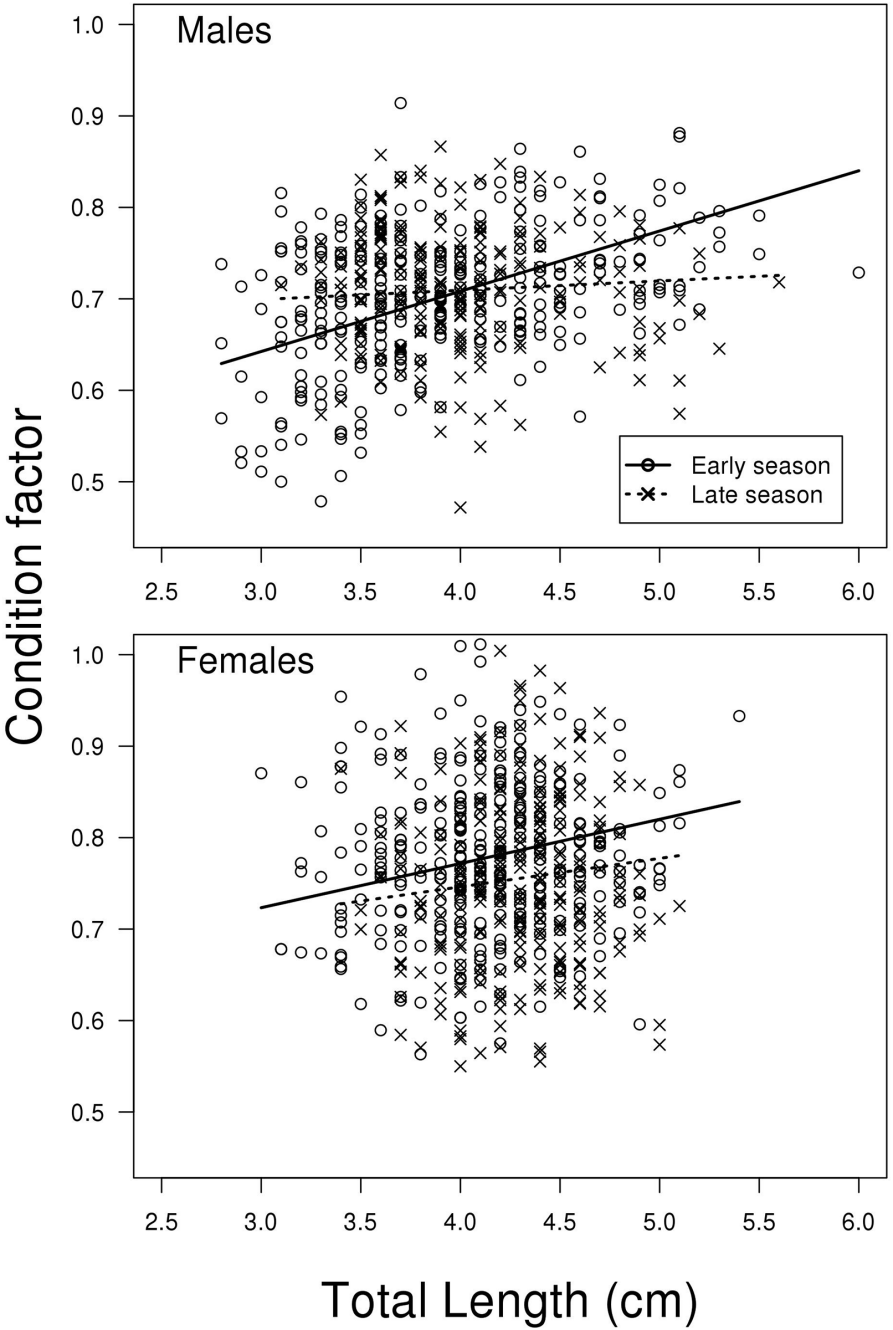
Condition factor. Total weight (g)*100/total length (cm)^3^) vs. length, for males sampled early and late in the reproductive season. N = 533.

The model for condition factor showed a strong tendency of a three-way interaction between season, size and sex (LME; F_1,1123_ = 3.742, P = 0.053, Fig 7). Thus, the change in slope depending on season is different between males and females. This is also seen in Fig 7 where the positive relationship between fish size and condition factor is clearly reduced when going from early to late season for the males but not for the females. The treatment contrasts of the model shows that the difference in slope of the two regression lines for females is not significant (t_1123_ = 1.079, P = 0.281, Fig 7), while it is significant for the males (same model but with relevelled treatment contrasts for sex; t_498_ = 4.712, P < 0.001, Fig 7). For a complete list of results from the model see Supporting information material (Model F in S1 File).

### Histology –presence of sperm in SDG related to size and season

**Fig 8:**
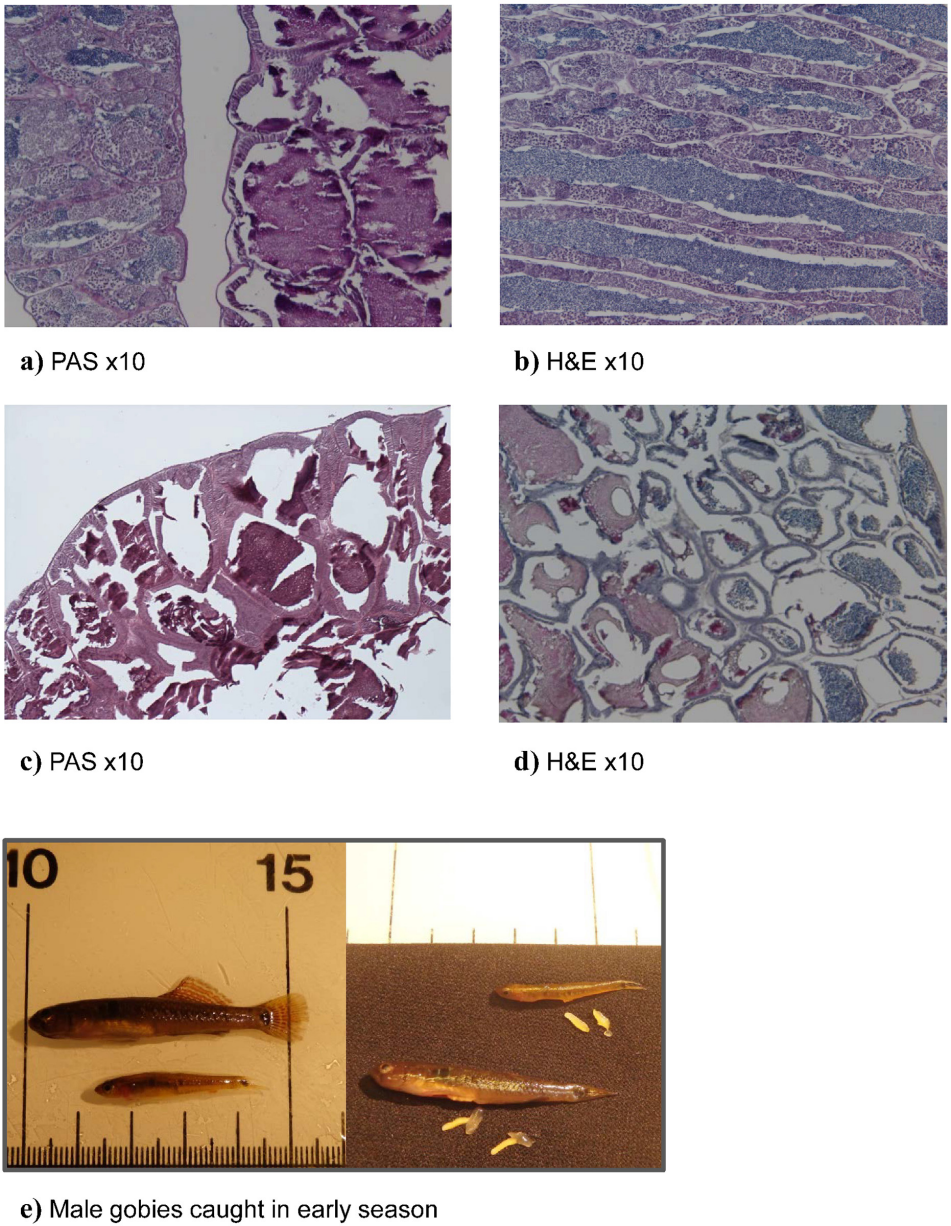
Cross sections of sperm duct gland and testes of male two-spotted goby, *Gobiusculus flavescens*. Testes (left) and seminal duct gland (right) of a 49 mm TL male goby sampled in the early breeding season (A). Staining with Periodic acid-Sciff`s (PAS). x10. Testes (B): spermatogonia and cysts of spermatocytes, secondary spermatocytes and spermatids are pressent in the lobule walls, while sperm fill lobule lumina. Male 34 mm TL from early in the breeding season. Haematoxylin and eosin staining. x10. Seminal duct gland (C): Chambers are highly extended and their lumina completely filled with mucin secretion. Male 41 mm TL from early in the breeding season. PAS. x10. Both epithelial cells and secretion react positively to PAS staining (Periodic acid-Sciff`s). Sperm duct gland of a 34 mm TL male goby, sampled in the early breeding season (D). Chambers are small poorly developed and many of them are filed with sperm and little or no secretion. Haematoxylin and eosin staining. x10. Pictures showing two males, caught in the early breeding season (May) with a total length of 53 and 33 mm, weight 0,840 and 0,120 g accordingly (E). Right picture shows same fish with dissected testes (yellow elongated) and SDG (transparent elongated). Testes and SDG weight of the 53 mm male were 0,0076 g and 0,0122 g, and for the 33 mm male 0,0100 and 0.0013 g accordingly (one of the SDG’s from the 33 mm male is missing on the picture).

All the 18 males whose reproductive organs were histologically examined were mature and had paired testes. The sperm transport system consisted of two main sperm ducts which fuse into a single common sperm duct, from which outgrow a pair of wing-like accessory structures (SDG) before reaching the urogenital opening. The testes are organized in lobules, of the unrestricted spermatogonial type [38, 39], whose walls were lined with germinal epithelium, presenting all the different stages of spermatogenesis, and lumina full of sperm in ripe males (Fig 8a). The SDGs are multi-chambered, as commonly observed in gobioid species [40]. The chamber wall consists of an internal single layer of epithelial cells, a basal lamina and an external thin layer of connective tissue (Fig 8a). The SDG’s chamber lumina were more or less full of a mucin-like substance, which reacted positively to staining for sialoglycoproteines (Fig 8, PAS a and c), and in some cases large amounts of sperm were also present in the lumina (Fig 8d). The quantities of mucin and sperm in the SDG differed in relation to size (TL). At the beginning of the breeding season in May, the four smallest males (30–34 mm TL) had small SDGs with a smaller lumina (0.40–0.56%) to chamber wall (0.60–0.44%) fraction of which 18–24% contained sperm (Table 1 and Fig 4d). In contrast, the eight intermediate and larger males caught in May had larger SDGs with large mucin-rich chambers containing no sperm (Table 1). Towards the end of the breeding season in July, all males had relatively large SDGs (see Fig 5) rich in mucin—giving a lumina fraction of 0.62–0.79. In the late breeding season smaller and medium sized males had sperm within 15–35% of the mucin-rich lumina chambers (Table 1). When grouping males into sneakers and territorial males based on histological findings (Table 1), we found that sneaker male’s invested more (higher “b” value, Table 2) in testes than in SDG early in the season, while the opposite was the case for territorial males. No such difference was found in late season, when all males invested most in SDG (Table 2).

**Table 1:** Length, GSI and SDGI values and histological data for male *Gobiusculus flavescens* collected in early (May) and late (July) reproduction season 2008.

Time	TL (mm)	GSI	SDGI	Sperm in SDG	AF Lumina	AF Chamber wall	% Lumina with sperm
**May**	30	3,8	0,7	Yes	0.40 ±0.10	0.60 ±0.10	0.24
	33	4,5	0,7	Yes	0.55 ±0.05	0.45 ±0.05	0.23
	33	5,1	0,7	Yes	NA	NA	NA
	34	7.2	1.0	Yes	0,45 ±0.09	0,55 ±0.09	0.18
	40	2.2	1.0	No	0.60 ±0.06	0.40 ±0.05	0
	41	3.6	2.7	No	0.70 ±0.02	0.30 ±0.02	0
	49	2.0	5.3	No	0.65 ±0.04	0.35 ±0.04	0
	49	2.8	1.9	No	0.64 ±0.09	0.36 ±0.05	0
	54	2.4	1.7	No	0.69 ±0.09	0.31 ±0.05	0
**July**	33	2.1	1.3	Yes	0.69 ±0.05	0.31 ±0.05	0.15
	33.5	3.4	2.0	Yes	0.70 ±0.06	0.30 ±0.05	0.22
	33.5	2.9	1.5	Yes	0.76 ±0.05	0.21 ±0.05	0.20
	35	2.8	1.5	Yes	0.79 ±0.04	0.21 ±0.04	0.32
	36	2.6	1.4	Yes	0.66 ±0.03	0.34 ±0.05	0.35
	42	1.7	1.1	Yes	0.62 ±0.15	0.38 ±0.05	0.30
	44	2.8	0.7	No	0.64 ±0.05	0.36 ±0.05	0
	47.5	2.0	0.6	No	0.59 ±0.05	0.41 ±0.05	0
	48.5	1.8	0.6	No	0.57 ±0.05	0.43 ±0.05	0

Male *Gobiusculus flavescens* collected in May and July 2008 presenting total length (TL) in mm gonadosomatic index (GSI; gonad weight/somatic weight *100) sperm duct gland index (SDGI; SDG weight/somatic weight *100) and whether histological study revealed presence of sperm in SDG or not. Areal fraction (AF) consisting of lumina or chamber wall is given as well as relative number (proportion) of luminas containing sperm.

**Table 2:** Allometric relations between seminal duct gland (SDG) and testes (T) weights vs soma weight for “sneaker” and “territorial” males categorised to tactics based on histological findings (Table 1) (early season: sneakers < 35 mm and territorial 40 mm <, late season: sneaker < 42 mm and territorial 44 mm <). Linear modelling was applied on log-transformed data to estimate the parameter b of the equation log(Y) = log(a) + b*log(X), where Y refers to SDG and Testes weight and X to soma weight. Standard error of estimate in parentheses. t-value and p-value are given for the estimated parameter b.

Tactic	N	ln(a) (± SE)	b (± SE)	t-value	p-value	df	R^2^
**Early season**							
**Sneaker: *SDG***	85	-0.88 (0.35)	0,91 (0.17)	5.27	<<0.001	84	0.25
***Testes***	85	-0.05 (0.11)	1.04 (0.05)	19.49	<<0.001	84	0.82
**Territorial: *SDG***	97	0.06 (0.15)	1.18 (0.09)	13.63	<<0.001	96	0.66
***Testes***	97	-0.88 (0.15)	0.71 (0.08)	8.45	<<0.001	96	0.43
**Late season**							
**Sneaker: *SDG***	190	0.50 (0.11)	1.34 (0.05)	4.76	<<0.001	189	0.79
***Testes***	190	-1.44 (0.15)	0,53 (0.07)	7.57	<<0.001	189	0.23
**Territorial: *SDG***	39	0.59 (0.11)	1,43 (0.06)	24.56	<<0.001	38	0.94
***Testes***	39	1.36 (0.13)	0.53 (0.07)	7.66	<<0.001	38	0.61

## Discussion

Here we report for the first time a population of two-spotted gobies in which males have a smaller average size (40 mm) compared to females (43 mm). All previous studies on two-spotted gobies (populations in Sweden, Scotland and Ireland) have reported the opposite, with males being larger than females, and further none of these studies reported the occurrence of small sneaker males. For example, on the Swedish west coast, which is the most thoroughly studied population, the gobies exhibit sexual size dimorphism, with males on average slightly larger than females (average male and female size being 47–48 and 42–47 mm respectively; [41, 42]. This indicates that the Bergen population is generally smaller, for both sexes, probably reflecting the lower water temperatures and shorter growing season for this more northerly population. There are few studies on intra specific variation in Sexual Size Dimorphism (SSD) in fish [43, 44]. However, these studies have revealed that SSD varies at least as much between fish populations of one species as it does between related species. Fishes plasticity in body size, together with intersexual competition that is affected by environmental variations, such as availability of nest sites or size related mortality is suggested explanations to the great variation in SSD among fish [43, 44].

As predicted, early in the breeding period the smaller males displayed typical sneaker morphological characteristics, with relatively large testes and small SDG’s containing sperm. We found that (1) testes weight decreased in all males size categories from early to late in the breeding season, with this decrease being more pronounced in the smaller and intermediate males (Fig 4). Accordingly, (2) the SDG weight increased significantly for smaller males from the early to late breeding season, which was not the case for larger and intermediate sized males (Fig 5). Further, (3) histological studies revealed the presence of sperm throughout large areas of the SDGs in small males both early and late in the breeding season, whereas the SDGs of larger males contained no sperm at any time of season (Table 1, Fig 8). This temporal shift found in the smaller males, from small sperm-rich SDGs to larger mucin-rich SDGs (still containing sperm) occurred concomitant with an overall decrease in testes investment (relative investment in SDG/ testes, Fig 6 and Table 2). This result basically implies a change in mating tactic among the smaller males. The sperm seen in the SDGs of small and intermediate sized males in the late breeding season could be sperm left over from a time when the SDGs functioned primarily for sperm storage (an earlier sneaking period), or that the SDGs continue to be used as sperm storage as well as mucin producers. The grass goby, *Zosterisessor ophiocephalus*, has been found to have the continuum type of seminal vesicles, with a decreasing amount of sperm with increasing body length [13]. In common with the black goby, *Gobius niger*, the grass goby was found to have an intermediate size-range of males who invested in both SDGs and gonads, and where the seminal vesicles were used for both sperm storage and mucin production [25]. This type of intermediate male (males that invest in SDG and store sperm there as well) may be too small to gain a nest when competing with larger males but are sufficiently large to compete opportunistically for vacated territories. Therefore depending on opportunity, they may become nesting males or adopt a sneaking tactic for mating [25]. These findings suggest that a territorial reproductive tactic is too costly for small males during the early breeding season, or females would not choose smaller males when male-male competition is high, and for these reasons the smaller males displayed an alternative sneaking tactic. However, our results also show that these smaller males increase their investment in SDG and mucin production towards the end of the season, indicating a lower male-male competition. A recent experimental study showed that if male two-spotted gobies are given limited access to mates (females) male-male competition increases, resulting in a positive selection towards male body size [45]. Thus, the present study showing a size effect on male reproductive tactic early, but not late in the breeding season, could be the result of an increase in female availability over the course of the season, as found in the Swedish population [19].

The change towards territorial type gonads for a broader size range of males is difficult to explain by higher mortality among the sneaker males. The demographic data does not indicate such a trend (Fig 1). As the positively skewed size distributions showed only minor changes throughout the sampling period this indicates that the mortality among smaller males is low. Only the intermediate size increase in numbers, indicating growth of smaller individuals, while the number of larger individuals decrease, presumably indicating mortality (also seen for females, Fig 1). A high mortality among nest-holding males compared to non-nest holders is likely [19], and is most probably caused by a combination of exhaustion resulting from parental care [22, 46], increased male—male competition [47], a higher susceptibility to parasites and diseases [48], and increased predation risk [49]. The small males seem to be very cryptic, as evidenced by the low number of small males (< 5% of males) observed when snorkeling (pers. observation), though being highly representative in the beach seine catches. Most likely, the smaller males remain hidden in the rocks and macro algae close to the nests of the dominant territorial males [17, 29], and as such are probably less exposed to predators.

Taken together, from the presented findings we suggest that the reproductive tactics of *G*. *flavescens* are conditional. Our findings indicate that the smaller, initially sneaker males can become nest holders or territorial males later in the breeding season. This switch in male reproductive tactic by smaller males could be the result of a number of changes occurring over the breeding season, such as a reduction in survival of eggs laid by larger males later in the season, possibly due to the more pronounced decline in condition factor in larger males (Fig 7), increasing the likelihood of egg cannibalism. Alternatively, as a consequence of a shift in the operational sex ratio (OSR) over the course of the breeding season, from initially a male to a female biased population (as reported in the Swedish population [19]), females now resort to courting smaller males (several females were seen courting small males in the late breeding season (July), while early in the breeding season (May) we only observed males courting females. pers. obs. A.C. Utne -Palm and M. Hordnes during a transect snorkeling survey at the same study sites, 2008). In contrast, such a size related tactic change over the breeding season was not found in the sand goby (*Pomatochistus minutus*) [36]. However, the authors of this study speculate that the tendency to observe fewer small colourless males late in the breeding season could be because these smaller males have changed mating tactics, from sneaker to the more colourful territorial males. Also the presented findings somewhat contradict a study of cuckoldry reported in a two-spotted goby population on the west coast of Sweden [29]. In their parental analysis of 21 nests collected during the breeding season, they found only one incidence of sneaking. This difference is somewhat unexpected given that this study was performed in the same location as an earlier study which reported a shift in operational sex ratio (OSR) from male to female biased population over the breeding season [18] with a following change from male to female courting over the season [21]. However, this study [29] was performed late in the breeding season.

The differences in testes and SDG investment in relation to size early in the breeding season could be explained by a change in tactic along an ontogenetic gradient. This type of plasticity in reproductive behaviour has been found in other gobies [12, 50, 51, 25]. Ontogeny could to some extent explain why we find predominantly small sized, presumably younger males (born late in season), with sneaker type gonads early in the breeding season, but which develop a more dominant gonad structure with older age later in the season. In a laboratory study on the black goby (*Gobius niger*), it was observed that young smaller sneaker males rapidly (within 3 to 4 weeks) changed their gonad investment from sneaker type to territorial type and developed male epigamic traits, when given exclusive access to a female and were free from competition by other males [14]. The same plasticity has been found in other fish species e.g. pupfish, *Cyprinodon pecosencis* [9]; longnose filefish, *Oxymonacanthus longirostris* [52] as well as other taxa e.g. American toad, *Bufo amencanus* [53] when skewing the OSR towards a female bias, both artificially [9, 14] and naturally [52]. A change in OSR, as found in other studies [19], or a change in relative physical condition factor (increasing c-factor of smaller individual and decreasing for larger individuals across season (Fig 7)) and relative size (status (Fig 1)) over the breeding season could be the main causal factor influencing the reproductive tactic of male two-spotted gobies on the west coast of Norway.

### Condition factor

In spadefoot toads (*Pelobates fuscus*) low body condition index was correlated with the use of alternative reproductive tactics [54]. This is in agreement with the presented findings, where we show an increase in condition factor with size early in the breeding season—while no such size relation is present in the late reproductive season for males (Fig 7). The latter can be interpreted as a result of the high energetic costs associated with the expression of secondary sexual traits and territorial reproductive behaviour [55, 56]. The negative effect of nest holding on the condition factor of males is further supported by a laboratory study on two-spotted goby [22] which showed that the condition factor of nest-holding males declines significantly while caring for eggs, whereas no change in condition factor was observed in non-nest holding males. In the present study there were more large males in the early compared to late breeding season (Fig 1). As numerous studies have shown a positive correlation between large body size and the adoption of the more energy demanding dominant reproductive strategy in gobies [12, 50, 51, 25], the reduction in size over the breeding season is likely explained by a higher mortality rate for larger nest-holding males, which also is supported by the demographic findings (Fig 1).

Overall, from data on relative testes and SDG investment, together with histological evidence, there is strong inferential data suggesting that the small males change their reproductive morphology through the season from sneaker to a more dominant nest-defending morphology. This appears to be in part due to the loss of larger males as the season progresses, possibly because of the higher energetic demands associated with parental care [22, 46]. Thus, taken together our results indicate that *G*. *flavescens* most likely has a conditional reproductive strategy.

Continued investment in somatic growth during the breeding season by initially smaller males allows these males to potentially adopt an opportunistic territorial tactic later in the breeding season when they have attained a better condition and/or larger size (Figs 1 and 7). Later in the breeding season, these smaller males should become more attractive to females, especially with the decline in CF and increased mortality among the larger males due to the energetic demands of brood caring. Which tactic offers the better reproductive success, and when a male should change between mating tactics (e.g. sneaker to territorial male) will depend on both the fitness of the dominant tactic and status of the male [53].

## Conclusion

The findings of this study support the theory that smaller males predominantly display a sneaking tactic early in the reproductive season when the costs associated with territoriality and the development of secondary sexual traits presumably are high. Further, changes seen in testes (GSI) and SDG investment (SDGI), and histological changes over the breeding season indicate that smaller males can take on a nest holding role as the breeding season proceeds. The latter being a possible response of: i) an increasing condition factor of smaller males and/or ii) an increasing female biased OSR over the season as a consequence of high mortality of territorial males. Thus, *G*. *flavescens* most likely has a conditional reproductive strategy.

## Supporting Information

S1 Dataset (CSV)

S1 File Statistical outputs from the following models are found in the Supporting information material. *Model A*, *GSI*. *Model B*, *SDGI*. *Model C*, *Testis*. *Model D*, *SDG*. *Model E*, *relative investment in SDG*. *Model F*, *condition factor*.
(DOC)
