# Supplementary material for: Size Dependent Male Reproductive Tactic in the Two-Spotted Goby (Gobiusculus flavescens)
Source: PLoS One. 2015 Dec 7;10(12):e0143487. doi: 10.1371/journal.pone.0143487 (PMC4674270; doi:10.1371/journal.pone.0143487)
Supplement: S1 File — Model A, GSI. Model B, SDGI. Model C, Testis. Model D, SDG. Model E, relative investment in SDG. Model F, condition factor. (DOC) [file pone.0143487.s002.doc]

**Supplementary material (S1):**

***Model A:***

*GSI*

**Table A:** Log likelihood ratio tests (LRT) for determining the number of curvature parameters (polynomial order) for the predictor variable total length. The first model (null model) only contains the intercept (+1) as predictor, while the ones listed below is showing how these differ from the null model, i.e. only the predictors of the models differ. All ΔAIC, LRT and P-values are comparison between a given model and the simpler model listed above it. A significant P-value means a significant improved model compared to the one above it, based on LRT. A negative ΔAIC-value also indicates an improved model compared to the one above it. Since the restricted maximum likelihood method (REML) is not possible when comparing these models, the maximum likelihod method (ML) were used.

R model syntax after determining the number of curvature parameters (polynomial order):

lme(GSI~poly(Total.length,2)*Season, random=~+1|Station, method='REML')

Model output:

numDF denDF F-value p-value

(Intercept) 1 498 343.7401 <.0001

poly(Total.length, 2) 2 498 287.0577 <.0001

Season 1 498 183.4088 <.0001

poly(Total.length, 2):Season 2 498 21.7634 <.0001

Summary output (treatment contrasts etc.):

Linear mixed-effects model fit by REML

Data: kutling.df

AIC BIC logLik

1140.012 1173.729 -562.0062

Random effects:

Formula: ~+1 | Station

(Intercept) Residual

StdDev: 0.1170079 0.7364545

Fixed effects: GSI ~ poly(Total.length, 2) * Season

Value Std.Error DF t-value p-value

(Intercept) 1.838807 0.0820256 498 22.417465 0.000

poly(Total.length, 2)1 -16.706545 0.9412870 498 -17.748620 0.000

poly(Total.length, 2)2 8.540125 0.9458212 498 9.029323 0.000

Seasonlate -0.983078 0.0689601 498 -14.255744 0.000

poly(Total.length, 2)1:Seasonlate 10.580847 1.6320626 498 6.483113 0.000

poly(Total.length, 2)2:Seasonlate -4.209782 1.7064786 498 -2.466941 0.014

Correlation:

(Intr) pl(T.,2)1 pl(T.,2)2 Sesnlt p(T.,2)1:

poly(Total.length, 2)1 0.013

poly(Total.length, 2)2 -0.115 0.134

Seasonlate -0.380 -0.023 0.137

poly(Total.length, 2)1:Seasonlate -0.012 -0.554 -0.083 -0.117

poly(Total.length, 2)2:Seasonlate 0.062 -0.065 -0.558 0.144 -0.195

Standardized Within-Group Residuals:

Min Q1 Med Q3 Max

-4.32807888 -0.38723880 -0.05670994 0.29932985 5.55144632

Number of Observations: 506

Number of Groups: 3

**Model B:**

*SDGI*

**Table B:** Log likelihood ratio tests (LRT) for determining the number of curvature parameters (polynomial order) for the predictor variable total length. See Table A for details about the content of the table and how it is used in model selection.

R model syntax after determining the number of curvature parameters (polynomial order):

lme(SDGI~poly(Total.length,2)*Season, random=~+1|Station, method='REML')

Model output:

numDF denDF F-value p-value

(Intercept) 1 498 471.1483 <.0001

poly(Total.length, 2) 2 498 12.5703 <.0001

Season 1 498 2.1321 0.1449

poly(Total.length, 2):Season 2 498 24.4928 <.0001

Summary output (treatment contrasts etc.):

Linear mixed-effects model fit by REML

Data: kutling.df

AIC BIC logLik

819.4166 853.1334 -401.7083

Random effects:

Formula: ~+1 | Station

(Intercept) Residual

StdDev: 0.0937776 0.534284

Fixed effects: SDGI ~ poly(Total.length, 2) * Season

Value Std.Error DF t-value p-value

(Intercept) 1.272729 0.0638050 498 19.947175 0.0000

poly(Total.length, 2)1 1.991002 0.6834438 498 2.913191 0.0037

poly(Total.length, 2)2 -4.292104 0.6863306 498 -6.253699 0.0000

Seasonlate 0.130731 0.0500296 498 2.613064 0.0092

poly(Total.length, 2)1:Seasonlate -4.687826 1.1840313 498 -3.959208 0.0001

poly(Total.length, 2)2:Seasonlate 7.967011 1.2382635 498 6.434019 0.0000

Correlation:

(Intr) pl(T.,2)1 pl(T.,2)2 Sesnlt p(T.,2)1:

poly(Total.length, 2)1 0.011

poly(Total.length, 2)2 -0.107 0.133

Seasonlate -0.355 -0.023 0.137

poly(Total.length, 2)1:Seasonlate -0.012 -0.553 -0.083 -0.117

poly(Total.length, 2)2:Seasonlate 0.058 -0.065 -0.558 0.144 -0.195

Standardized Within-Group Residuals:

Min Q1 Med Q3 Max

-2.2343977 -0.7086769 -0.1651498 0.5823600 3.8556485

Number of Observations: 506

Number of Groups: 3

**Model C:**

*Testis*

**Table C:** Log likelihood ratio tests (LRT) for determining the number of curvature parameters (polynomial order) for the predictor variable log Soma mass. See Table A for details about the content of the table and how it is used in model selection.

R model syntax after determining the number of curvature parameters (polynomial order):

lme(log.Testis.mass~poly(log.Soma.mass,5)*Season, random=~+1|Station, method='REML')

Model output:

numDF denDF F-value p-value

(Intercept) 1 492 9402.731 <.0001

poly(log.Soma.mass, 5) 5 492 52.855 <.0001

Season 1 492 384.107 <.0001

poly(log.Soma.mass, 5):Season 5 492 3.048 0.0101

Due to high complexity (high number of curvature parameters), the model was replaced by the following GAM:

gam(log.Testis.mass~s(log.Soma.mass) + factor(Season) + s(log.Soma.mass, by=Season), family='gaussian')

Model output:

Family: gaussian

Link function: identity

Formula:

log.Testis.mass ~ s(log.Soma.mass) + factor(Season) + s(log.Soma.mass,

by = Season)

Parametric coefficients:

Estimate Std. Error t value Pr(>|t|)

(Intercept) -2.20674 0.01033 -213.69 <2e-16 ***

factor(Season)late -0.28934 0.01687 -17.15 <2e-16 ***

---

Signif. codes: 0 ‘***’ 0.001 ‘**’ 0.01 ‘*’ 0.05 ‘.’ 0.1 ‘ ’ 1

Approximate significance of smooth terms:

edf Ref.df F p-value

s(log.Soma.mass) 0.6667 0.6667 5.776 0.0503 .

s(log.Soma.mass):Seasonearly 4.9116 6.0554 9.741 3.02e-10 ***

s(log.Soma.mass):Seasonlate 2.8381 3.6687 9.433 1.63e-06 ***

---

Signif. codes: 0 ‘***’ 0.001 ‘**’ 0.01 ‘*’ 0.05 ‘.’ 0.1 ‘ ’ 1

Rank: 28/29

R-sq.(adj) = 0.558 Deviance explained = 56.6%

GCV = 0.026382 Scale est. = 0.025839 n = 506

**Model D:**

*SDG*

**Table D:** Log likelihood ratio tests (LRT) for determining the number of curvature parameters (polynomial order) for the predictor variable Total length. See Table A for details about the content of the table and how it is used in model selection.

R model syntax after determining the number of curvature parameters (polynomial order):

lme(log.Seminal.duct.gland.mass~poly(log.Soma.mass,2)*Season, random=~+1|Station, method='REML')

Model output:

numDF denDF F-value p-value

(Intercept) 1 498 5162.131 <.0001

poly(log.Soma.mass, 2) 2 498 251.135 <.0001

Season 1 498 0.004 0.9478

poly(log.Soma.mass, 2):Season 2 498 20.625 <.0001

Summary output (treatment contrasts etc.):

Summary output (treatment contrasts etc.):

Linear mixed-effects model fit by REML

Data: kutling.df

AIC BIC logLik

-16.37808 17.33879 16.18904

Random effects:

Formula: ~+1 | Station

(Intercept) Residual

StdDev: 0.05323299 0.2315503

Fixed effects: log.Seminal.duct.gland.mass ~ poly(log.Soma.mass, 2) * Season

Value Std.Error DF t-value p-value

(Intercept) -2.330302 0.0340918 498 -68.35377 0.0000

poly(log.Soma.mass, 2)1 5.728903 0.2836022 498 20.20049 0.0000

poly(log.Soma.mass, 2)2 -1.644178 0.2826454 498 -5.81710 0.0000

Seasonlate 0.052048 0.0232455 498 2.23907 0.0256

poly(log.Soma.mass, 2)1:Seasonlate -3.258811 0.5882491 498 -5.53985 0.0000

poly(log.Soma.mass, 2)2:Seasonlate 3.452956 0.6647479 498 5.19438 0.0000

Correlation:

(Intr) pl(.S.,2)1 pl(.S.,2)2 Sesnlt

poly(log.Soma.mass, 2)1 0.025

poly(log.Soma.mass, 2)2 -0.098 0.097

Seasonlate -0.273 -0.038 0.141

poly(log.Soma.mass, 2)1:Seasonlate -0.015 -0.478 -0.044 -0.254

poly(log.Soma.mass, 2)2:Seasonlate 0.040 -0.024 -0.435 0.319

p(.S.,2)1:

poly(log.Soma.mass, 2)1

poly(log.Soma.mass, 2)2

Seasonlate

poly(log.Soma.mass, 2)1:Seasonlate

poly(log.Soma.mass, 2)2:Seasonlate -0.400

Standardized Within-Group Residuals:

Min Q1 Med Q3 Max

-7.12755188 -0.43899795 0.08493955 0.56279853 2.28497452

Number of Observations: 506

Number of Groups: 3

**Model E:**

*Relative investment in SDG*

**Table E:** Log likelihood ratio tests (LRT) for determining the number of curvature parameters (polynomial order) for the predictor variable log Soma mass. See Table A for details about the content of the table and how it is used in model selection.

R model syntax after determining the number of curvature parameters (polynomial order):

lme(rel.invest~poly(log.Soma.mass,4)*Season, random=~+1|Station, method='REML')

Model output:

numDF denDF F-value p-value

(Intercept) 1 494 0.07754 0.7808

poly(log.Soma.mass, 4) 4 494 125.64018 <.0001

Season 1 494 130.12708 <.0001

poly(log.Soma.mass, 4):Season 4 494 9.98881 <.0001

Due to high complexity (high number of curvature parameters), the model was replaced by the following GAM:

gam(rel.invest~s(log.Soma.mass) + factor(Season) + s(log.Soma.mass, by=Season), family='gaussian')

Model output:

Family: gaussian

Link function: identity

Formula:

rel.invest ~ s(log.Soma.mass) + factor(Season) + s(log.Soma.mass,

by = Season)

Parametric coefficients:

Estimate Std. Error t value Pr(>|t|)

(Intercept) -0.11437 0.01755 -6.518 1.76e-10 ***

factor(Season)late 0.32654 0.02677 12.200 < 2e-16 ***

---

Signif. codes: 0 ‘***’ 0.001 ‘**’ 0.01 ‘*’ 0.05 ‘.’ 0.1 ‘ ’ 1

Approximate significance of smooth terms:

edf Ref.df F p-value

s(log.Soma.mass) 1.9965 2.5993 0.728 0.515

s(log.Soma.mass):Seasonearly 3.9988 5.0076 5.600 4.85e-05 ***

s(log.Soma.mass):Seasonlate 0.6667 0.6667 0.515 0.558

---

Signif. codes: 0 ‘***’ 0.001 ‘**’ 0.01 ‘*’ 0.05 ‘.’ 0.1 ‘ ’ 1

Rank: 28/29

R-sq.(adj) = 0.544 Deviance explained = 55.1%

GCV = 0.076192 Scale est. = 0.074887 n = 506

**Model F:**

*Condition factor*

R model syntax:

lme(C.factor~Season*Total.length, random=~+1|Station)

#The same model but with relevelled treatment contrasts for Sex:

Sex2 <- relevel(Sex, ref='male')

lme(C.factor~Sex2*Season*Total.length, random=~+1|Station)

Model output:

numDF denDF F-value p-value

(Intercept) 1 1123 711.6530 <.0001

Sex 1 1123 215.9291 <.0001

Season 1 1123 3.7593 0.0528

Total.length 1 1123 97.9838 <.0001

Sex:Season 1 1123 16.9233 <.0001

Sex:Total.length 1 1123 0.4126 0.5208

Season:Total.length 1 1123 19.6365 <.0001

Sex:Season:Total.length 1 1123 3.7419 0.0533

Summary output (treatment contrasts etc.):

Linear mixed-effects model fit by REML

Data: NULL

AIC BIC logLik

-2723.08 -2672.825 1371.54

Random effects:

Formula: ~+1 | Station

(Intercept) Residual

StdDev: 0.04774598 0.06996146

Fixed effects: C.factor ~ Sex * Season * Total.length

Value Std.Error DF t-value p-value

(Intercept) 0.5783682 0.04803177 1123 12.041369 0.0000

Sexmale -0.1335502 0.04665617 1123 -2.862435 0.0043

Seasonlate 0.0434572 0.06777546 1123 0.641194 0.5215

Total.length 0.0483356 0.00949059 1123 5.093007 0.0000

Sexmale:Seasonlate 0.1805351 0.08253891 1123 2.187273 0.0289

Sexmale:Total.length 0.0175377 0.01144174 1123 1.532781 0.1256

Seasonlate:Total.length -0.0172436 0.01598372 1123 -1.078825 0.2809

Sexmale:Seasonlate:Total.length -0.0384740 0.01988927 1123 -1.934412 0.0533

Correlation:

(Intr) Sexmal Sesnlt Ttl.ln Sxml:S Sxm:T.

Sexmale -0.676

Seasonlate -0.463 0.475

Total.length -0.815 0.821 0.562

Sexmale:Seasonlate 0.382 -0.566 -0.822 -0.463

Sexmale:Total.length 0.661 -0.992 -0.464 -0.811 0.562

Seasonlate:Total.length 0.471 -0.483 -0.996 -0.579 0.819 0.478

Sexmale:Seasonlate:Total.length -0.380 0.572 0.801 0.466 -0.994 -0.576

Ssn:T.

Sexmale

Seasonlate

Total.length

Sexmale:Seasonlate

Sexmale:Total.length

Seasonlate:Total.length

Sexmale:Seasonlate:Total.length -0.804

Standardized Within-Group Residuals:

Min Q1 Med Q3 Max

-3.98795743 -0.65882095 -0.01573441 0.67794057 3.60195062

Number of Observations: 1133

Number of Groups: 3

The parameter estimates of the same model but with relevelled treatment contrasts for Sex, i.e. with males before females:

Fixed effects: C.factor ~ Sex2 * Season * Total.length

Value Std.Error DF t-value p-value

(Intercept) 0.4448180 0.03815046 1123 11.659572 0.0000

Sex2female 0.1335502 0.04665617 1123 2.862435 0.0043

Seasonlate 0.2239923 0.04704562 1123 4.761173 0.0000

Total.length 0.0658733 0.00669940 1123 9.832713 0.0000

Sex2female:Seasonlate -0.1805351 0.08253891 1123 -2.187273 0.0289

Sex2female:Total.length -0.0175377 0.01144174 1123 -1.532781 0.1256

Seasonlate:Total.length -0.0557177 0.01182371 1123 -4.712368 0.0000

Sex2female:Seasonlate:Total.length 0.0384740 0.01988927 1123 1.934412 0.0533
